# Comparison of diffusion kurtosis imaging versus diffusion weighted imaging in predicting the recurrence of early stage single nodules of hepatocellular carcinoma treated by radiofrequency ablation

**DOI:** 10.1186/s40644-019-0213-9

**Published:** 2019-05-29

**Authors:** Zhen-Guo Yuan, Zong-Ying Wang, Meng-Ying Xia, Feng-Zhi Li, Yao Li, Zhen Shen, Xi-Zhen Wang

**Affiliations:** 1 Medical Imaging Center of the Affiliated Hospital, Weifang Medical University, Weifang, 261053 People’s Republic of China; 2 Shandong Medical Imaging Research Institute Affiliated to Shandong University, Jinan, 250021 People’s Republic of China

**Keywords:** Diffusion kurtosis imaging, Diffusion weighted imaging, Hepatocellular carcinoma, Radiofrequency ablation

## Abstract

**Objective:**

This study aimed to compare the diffusion kurtosis imaging (DKI) versus diffusion weighted imaging (DWI) in predicting the recurrence of early stage single nodules of hepatocellular carcinoma (HCC) treated by radiofrequency ablation (RFA).

**Materials and methods:**

A retrospective analysis of 107 patients with early stage single nodules of HCC was performed, all patients treated by RFA. Recurrence rate of HCC was recorded after a median follow-up of 36 months. During follow-up, the data of magnetic resonance imaging (MRI), DWI and DKI were obtained in multiple time points. The predictive values of DWI and DKI were analyzed using ROC curves.

**Results:**

The overall recurrence rate was 66.3% (71/107). The sensitivity, specificity, and AUC for ADC, MD and MK after RFA (78.6, 73.3% and 0.842; 85.7, 83.3% and 0.839; 85.7, 96.7% and 0.956) were higher than before RFA (44.3, 53.3% and 0.560; 51.2, 56.7% and 0.543; 43.6, 67.3% and 0.489). The sensitivity, specificity, and AUC for MK after RFA were 85.7, 96.7%, and 0.956, respectively, which were significantly greater than those of ADC (78.6, 73.3% and 0.842; *P* < 0.05) and MD (85.7, 83.3% and 0.839).

**Conclusions:**

The prediction efficacy of DKI for the recurrence of early stage single nodules of HCC was better than that of DWI. And, MK was the most sensitive predictor among the DKI.

## Introduction

Hepatocellular carcinoma (HCC) is a major health problem worldwide due to its high incidence (more than 500,000 cases per year) and poor prognosis [1]. Early stage single nodules of HCC were defined as single nodules with a maximum diameter of less than 3.0 cm. Radiofrequency ablation (RFA) is assumed to be a suitable treatment option for HCC, especially for early stage HCCs and patients who are unsuitable for surgery [2, 3]. A study revealed that the five-year overall survival rate of patients who received RFA as the preferred treatment for HCC is approximately 60% [4]. The peri-operative mortality of RFA is junior to that of surgical excision, however, the recurrence rate is higher than that of surgical resection. Several biological factors, such as microRNA-34a and microvascular invasion (MVI), were important predictors to evaluate the prognosis of HCC [5, 6]. However, during follow-up, finding an effective and non-invasive method is necessary to evaluate the prognosis of HCC.

Over the last decades, several studies on functional magnetic resonance imaging, such as diffusion-weighted imaging (DWI), have demonstrated the value in diagnosis and preoperative and postoperative evaluation of HCC [7]. Tumors were frequently more cellular than the tissue from which they originate, and the diffusion of water molecules was restricted, thus resulting in relatively higher signal intensity on DWI [8]. Studies have shown that DWI was mainly applied to quantify the diffusion of water molecules with Gaussian distribution [9]. The homogeneous or single water molecules diffusion movement was called Gaussian motion [10]. As a matter of fact, water diffusion in biologic tissues is restricted by its interactions with other molecules and cell membranes, which was named as non-Gaussian water diffusion. Therefore, DWI may be inadequate to describe the actualdiffusion in HCC [11]. Diffusion kurtosis imaging (DKI) was emerged as a promising technology. Jensen first proposed the concept of DKI at New York University in 2005 [11]. DKI provides novel in vivo diffusion properties that describe the tissue microstructure by analyzing diffusivity and kurtosis, which is a unitless index of non-Gaussianity. So, DKI could detect the water molecule diffusion in tissues with non-Gaussian distribution and reflect the microscopic changes of HCC. A study on clear cell renal cell carcinoma revealed that DKI can evaluate the characteristics of tumors and reflect the microstructural changes and complexity of tumor tissues [12].

At present, studies on the evaluation of HCC by DKI were fewer. Thus, our study aimed to compare DKI versus DWI in predicting the recurrence of early stage single nodules of hepatocellular carcinoma treated by radiofrequency ablation.

## Materials and methods

### Patients

**Fig. 1:**
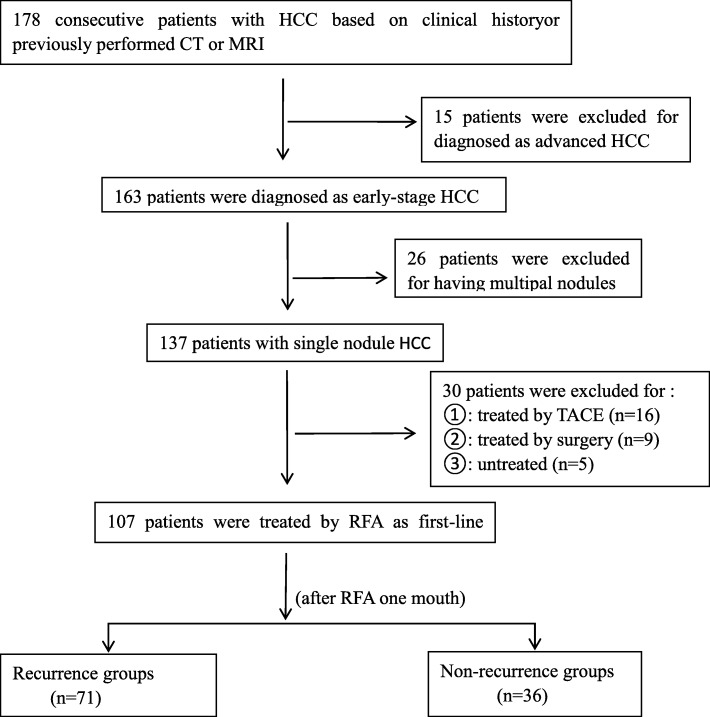
Follow-up flow chart

This study was approved by the local institute review board, and each patient signed the written informed consent. 178 consecutive patients with HCC based on clinical history or previously performed MRI between December 2016 and January 2018 in my hospital were recruited. We excluded 15 patients for they were been diagnosed as advanced HCC. 163 patients were diagnosed as early stage HCC. 26 patients were excluded with multiple nodules. Among the 137 patients with single nodules, 30 patients were excluded from our study population for the following reasons: 16 patients were treated by transcatheter arterial chemoembolization (TACE), 9 patients were treated by surgical therapies and 5 patients were untreated. Radiofrequency ablation was the first choice for 107 patients (Fig. 1). 107 subjects fulfilled with the following inclusion criteria: [1] 1 ≤ single HCC ≤ 3 cm without portal venous thrombosisor metastasis (BCLC 0/A), [2] compensated cirrhosis (Child-Pugh A or B), [3] no others treatment for HCC. Accordingly, among the 107 patients, 74 were males and 33 were females. The age range is between 42 and 86 years. The average age is 63 years. Clinical characteristics of the patients and tumors analyzed in this study are summarized in Table 1.

**Table 1:** Clinical characteristic of the patients and tumors

	Numbers
Male/female	74/33
Age (yr)	mean 63, range 42–86
Liver cirrhosis (*n*)	98
Hepatitis B (*n*)	83
Child-Pugh A/B (*n*)	95/12
Serum AFP levels, ng/ml, mean ± SD	73.3 ± 281.2
HCC median size, cm	1. 81 ± 0.83
BCLC stage 0/A (*n*)	87/20
Edmondson grade 3 or 4 (*n*)	28

Note-Available in 107patients

### Examination methods

Conventional MRI and DKI examinations of the liver were performed on all subjects before RFA and one month later after RFA, then followed-up every 3 months. The study was performed with a 3.0 T magnetic resonance imaging (MRI) scanner (Achieva, Philips Healthcare, Best, Netherlands). A standard 18-channel mode was employed for the body phased array coil. The regular MRI scan sequence included axial T1WI, axial fat-saturated T2WI, axial DWI sequence (b = 0, 800 mm^2^/s), and three-phase dynamic contrast-enhanced (DCE) and DKI sequence (b = 0, 800, 1500, 2000 mm^2^/s). All scan parameters were displayed in the Table 2.

**Table 2:** Protocols of MR sequences

	axial T1WI	axial STIR- T2WI	axial contrast-enhanced imaging (THRIVE)	axial DWI	axial DKI
TR/TE (ms)	206/3.83	3500/87	6.75/2.39	4800/64	3300/88
Flip angle	65°	150°	10°	90	90°
Field of view (mm^2^)	380 × 380	380 × 380	380 × 380	380 × 380	380 × 420
Slicethickness (mm)	6	6	6	6	5
Slice interval (mm)	2	2	2	2	1.5
Number of slices	26	26	72	26	26
NEX	1	3	1	4	3
b values (s/mm^2^)				0,800	1800,1500,2000

Note- *NEX* number of excitations, *SPIR* spectral fat saturation inversion recovery, *THRIVE* three-dimensionalT1WI fast field-echo high-resolution isotropic volume examination

### RFA procedures

RFA was implemented by using internally cooled electrodes with an exposed tip (Cool-tip RFA system, Valleylab, Boulder, CO, USA). Depending on the tumor size, location and the relationships with surrounding organizations, ablation was undertaken by the percutaneous route, laparoscopic or open approach. The procedure was done by an experienced interventional radiologist. It was effective in producing complete tumor necrosis, with ablation of a margin of non-tumors tissue of 1 cm after RFA. All patients had complete ablation for the treated lesions by RFA. MRI was performed to evaluate the treatment efficacy after RFA procedure. Multiple overlapping ablations were performed in order to achieve an adequate ablation if needed.

### Following up

Each subject performed contrast-enhanced MRI and DKI after initiating RFA one month, and then followed-up every 3 months. In this study, one month was considered as the earliest time point to assess the tumors, which may guide timely decision-making for subsequent therapies. The HCCs were classified as either recurrence groups or non-recurrence groups, which was assessed according to the overall mRECIST [13]. Non-recurrence groups were classified as complete necrosis, partial necrosis and stable nodules. Recurrence groups were defined as the sum of the longest diameters of the target tumors increased greater than 20% after RFA. Figure 1 represents the flow chart of HCCs.

### Data post-processing

The original data was transferred to PHILIPS workstation synchronously and post-processed by MRI Toolbox software package. According to DKI theories, S(b) = S_0_·exp. (−b·D + b^2^·D^2^·K/6), where S and S_0_ was the signal intensity of the images acquired at b and b_0_, respectively (s/mm^2^), and b is the b-value (s/mm^2^); D is the corrected apparent diffusion parameter of the Gaussian distribution, and K is the apparent kurtosis coefficient. DKI parameters (MD, MK) were recorded using b values (b = 0, 800, 1500, and 2000 s/mm^2^). Standard ADC (mm^2^/s) was obtained using a conventional mono-exponential fit with the following equation: S(b) = S_0_·exp.(−b·ADC). The ADC was calculated using b values of 0 and 800 s/mm^2^. The parameters were measured three times. The region of interest (ROI) was then selected. Two radiologists with different years of experience in abdominal imaging independently placed ROIs for each lesion. The principle of ROI selection were as follows: ① combined with conventional T1WI and T2WI images, the large lesion dimension was set as the ROI, where the scope of the lesion should be as large as possible; ②the internal areas that were necrotic or adjacent to blood vessels or bile ducts were excluded; ③the lesion ROIs were set at three different positions for each case to reduce error and the selected range was consistently maintained for each patient; ④ the maximum diameter of the HCC was measured on an axial T2WI on the basis of the reference T1WI enhanced image. The ROI of the lesions ranged from 1.0 cm^2^ to 2.5cm^2^. Statistical analyses were performed to compare the results of clinical and laboratory examinations. The differences in the corresponding parameters and the correlation with HCC prognosis were analyzed.

### Statistical analysis

Data obtained from DKI and ADC was statistically analyzed with SPSS 20.0 statistics software (IBMCorp, Armonk, NY, USA). Quantitative data were expressed as the mean ± standard deviation. ADC, MK, and MD of pre-RFA and post-RFA were analyzed using paired *t*-test. The sensitivity, specificity, and AUC under the ROC curve were calculated using MedCalc software (version11.4.2.0).

## Results

**Fig. 2:**
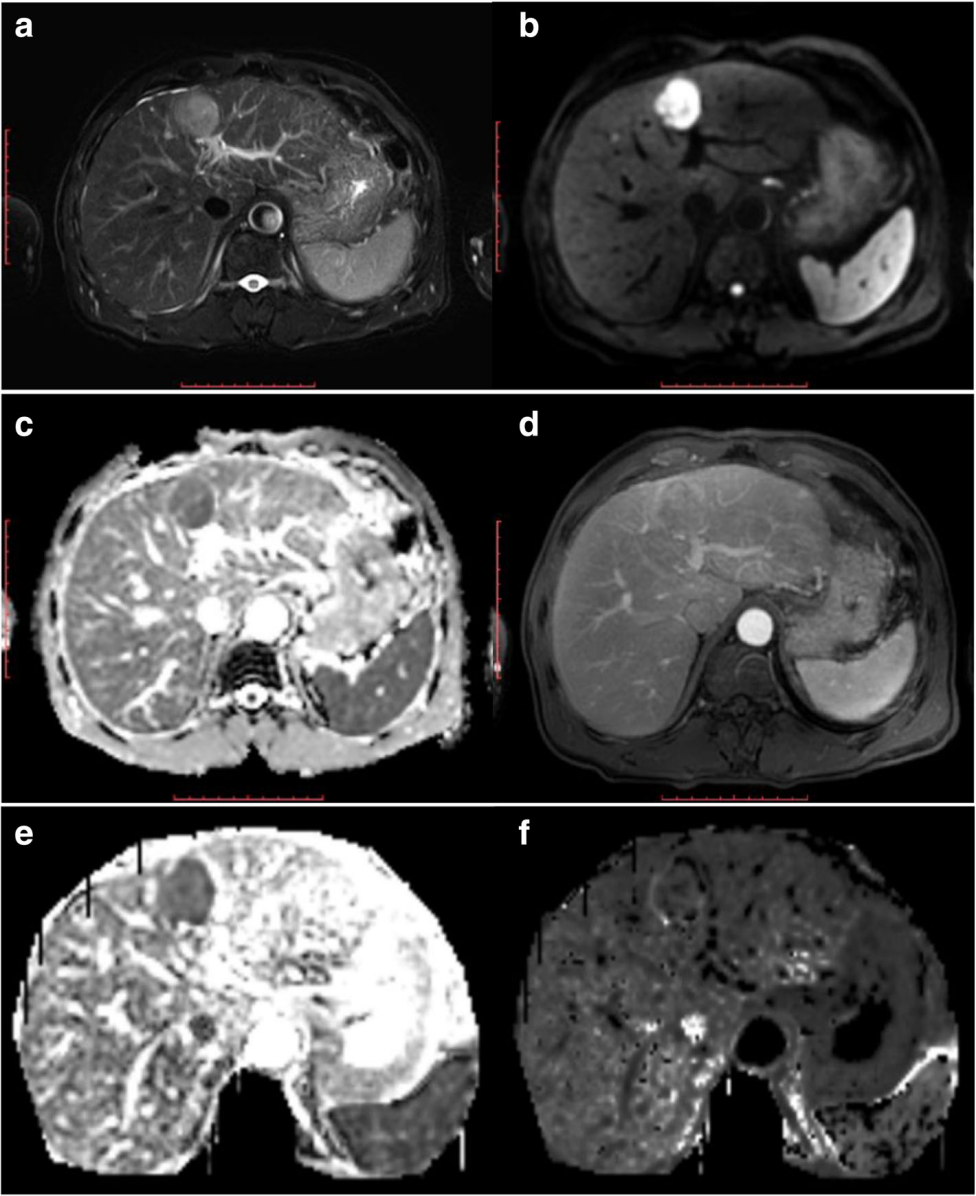
Images of a 48-year-old man with early stage single nodule of HCC. **a** axial fat-saturated T2WI image shows that the lesion of left hepatic lobe is slightly high signal intensity. **b** and **c** axial DWI and ADC map indicate that the diffusion of the lesion is obviously limited. **d** DCE arterial phase shows that the lesion has mild enhancement. **e** DKI-MD map shows that the MD value for the lesion is 1.181 × 10^− 3^. **f** DKI-MK map shows that the MK value for the lesion is 7.721 × 10^− 1^

**Fig. 3:**
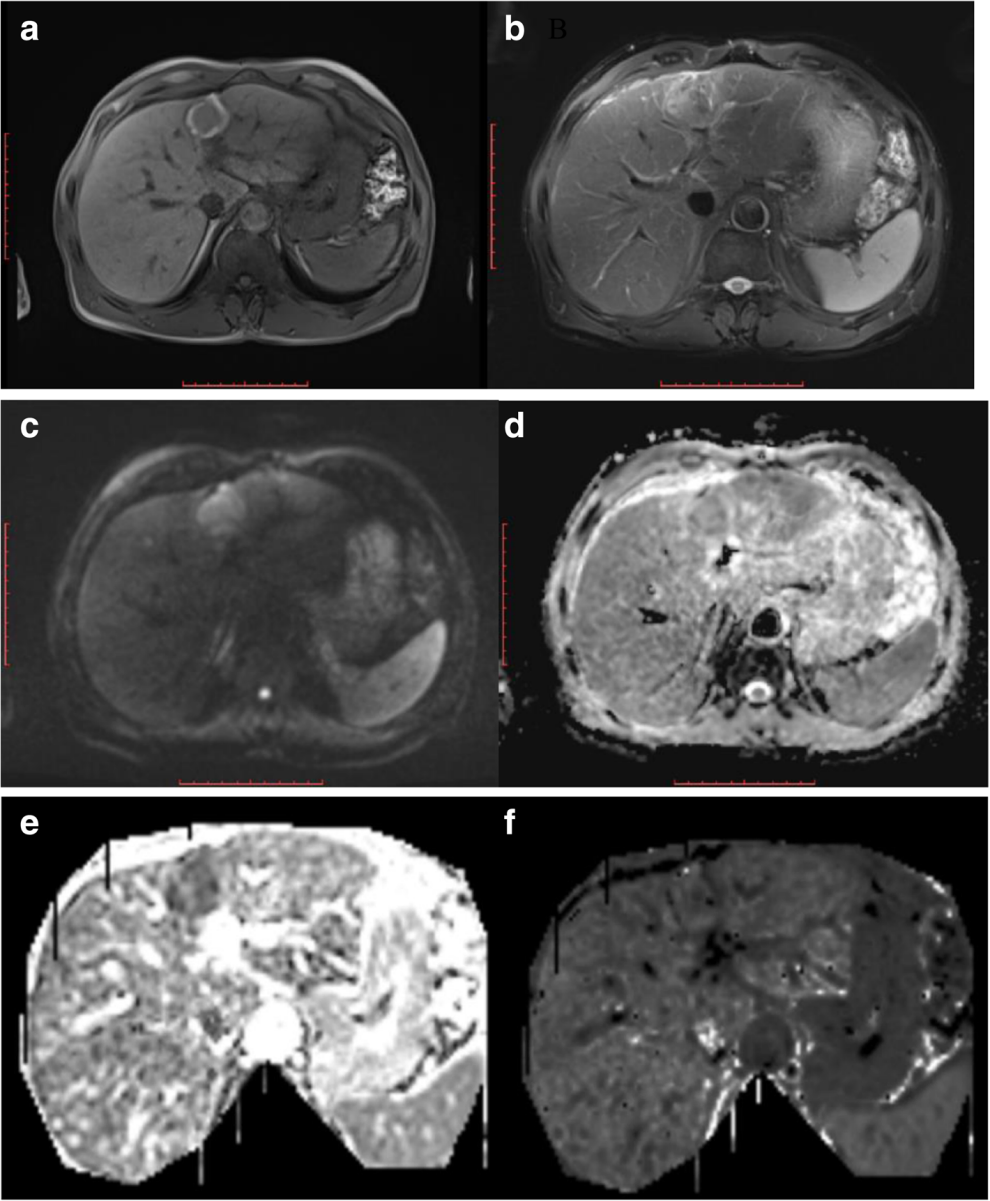
(The same patient in Fig. 2, after treatment by RFA for one month) **a** axial T1WI and **b** axial fat-saturated T2WI indicate that the signal strength of the lesion is reduced compared with that before. **c** and **d**: axial DWI and ADC map show that the diffused degree of the lesion is obviously reduced. **e** DKI-MD map show that the MD value for the lesion is 1.300 × 10^− 3^. **f** DKI-MK map show that the MK value for the lesion is 5.906 × 10^− 1^

**Fig. 4:**
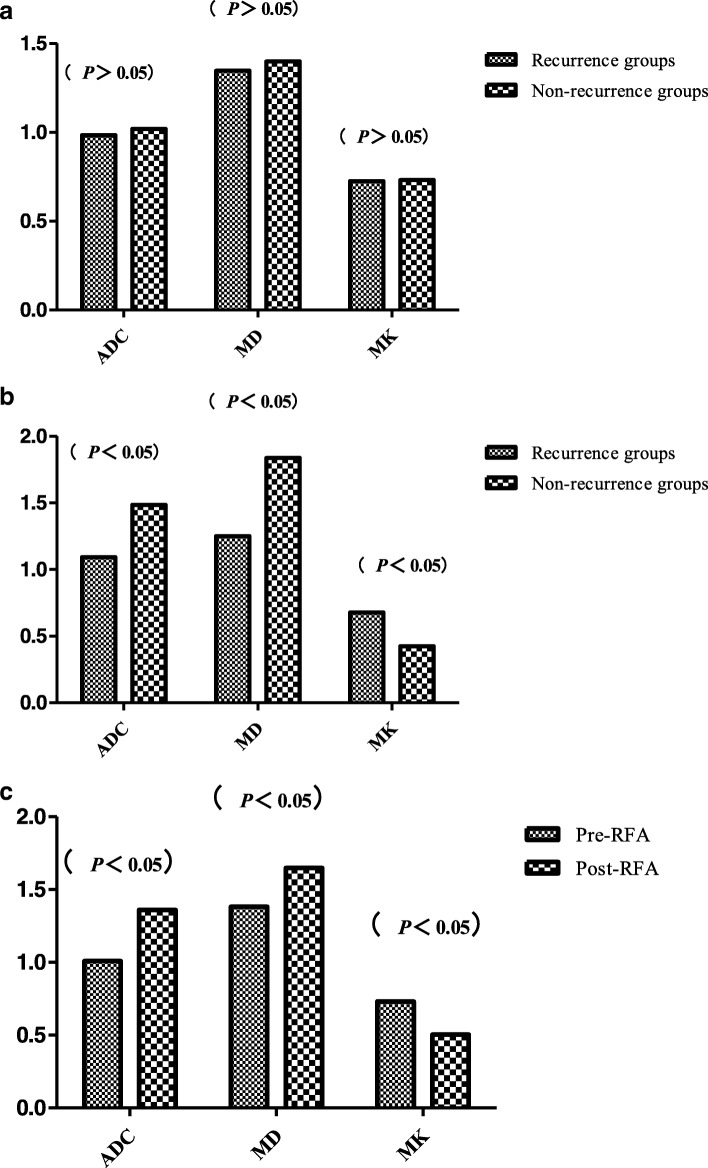
**a** The ADC, MD and MK of the recurrence groups and Non-recurrence groups before RFA (*P>*0.05). **b** The ADC, MD and MK of the recurrence groups and Non-recurrence groups after RFA (*P*<0.05). **c** The values of ADC, MD and MK before and after RFA (*P* < 0.05)

After a median follow-up of 36 months (range of 2–58 months), among the 107 HCCs treated by RFA, 36 HCCs were classed as non-recurrence groups. Among the 36 HCC nodules, 27 HCCs were complete necrosis, 7 HCCs were partial necrosis and 2 HCCs were stable disease. The recurrence rate was 66.3% (71/107). No obvious differences were observed in ADC, MD, and MK of pre-RFA between the recurrence (0.98 × 10^− 3^ mm^2^/s, 1.347 × 10^− 3^ mm^2^/s, 0.726) and non-recurrence groups (1.020 × 10^− 3^ mm^2^/s, 1.400 × 10^− 3^ mm^2^/s, 0.732) (*P* > 0.05, Fig. 4a). However, postoperative correlation parameter values were statistically significant (*P* < 0.05). The ADC and MD values of the recurrence groups (1.092 × 10^− 3^ mm^2^/s, 1.251 × 10^− 3^ mm^2^/s) were lower than those of the non-recurrence groups (1.486 × 10^− 3^ mm^2^/s, 1.837 × 10^− 3^ mm^2^/s) after RFA, the MK of the recurrence groups (0.678) was higher than that of the non-recurrence groups (0.424)(*P* < 0.05, Fig. 4b). Whether the recurrence groups or the non-recurrence groups, after RFA, the values of ADC and MD were higher (1.361 × 10^− 3^ mm^2^/s, 1.650 × 10^− 3^ mm^2^/s) than pre-RFA (1.009 × 10^− 3^ mm^2^/s, 1.382 × 10^− 3^ mm^2^/s); MK value was lower after RFA (0.505) than pre-RFA (0.732) (*P* < 0.05, Figs. 2, 3, 4c).

**Fig. 5:**
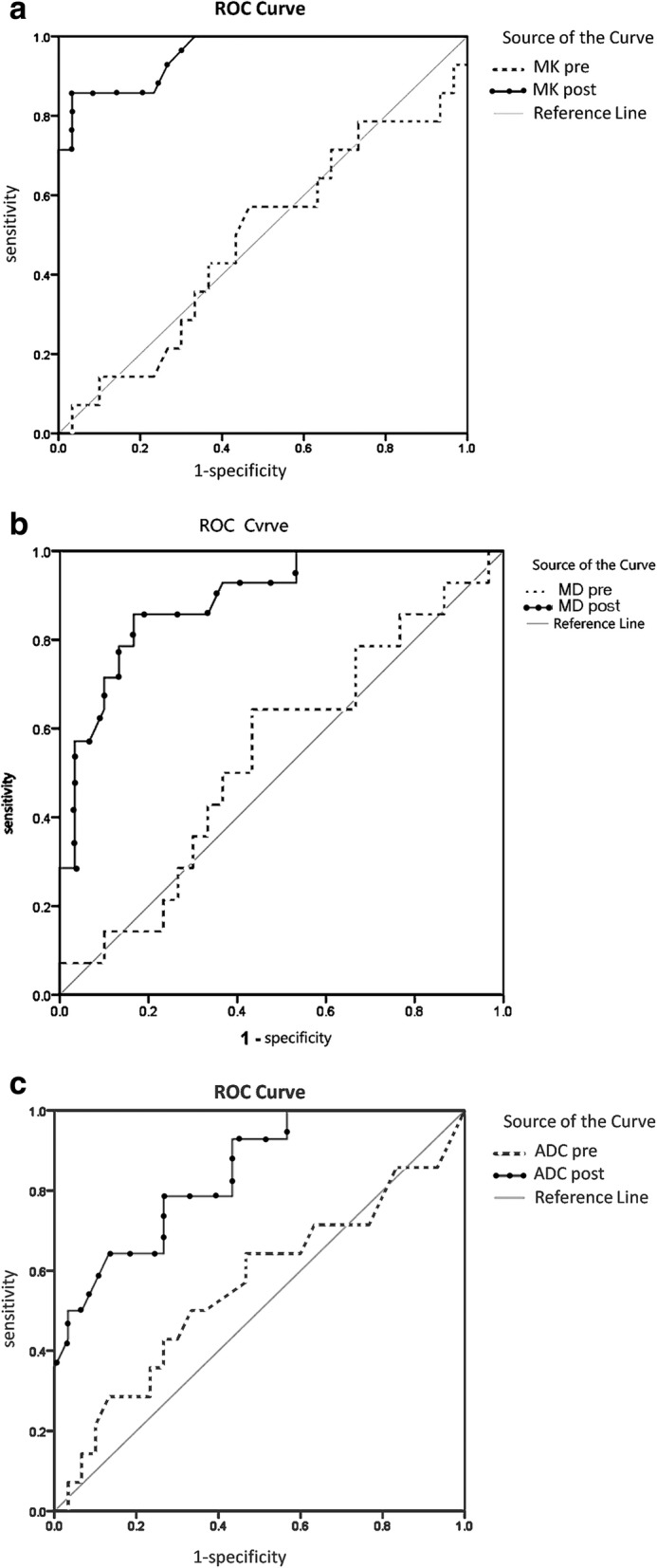
**a**: ROC curve of MK. **b** ROC curve of MD. **c** ROC curve of ADC

The sensitivity, specificity, and area under the curve (AUC) for ADC, MD and MK after RFA (78.6, 73.3% and 0.842; 85.7, 83.8% and 0.839; 85.7, 96.7% and 0.956) were higher than before RFA (44.3, 55.3% and 0.560; 51.2, 56.7% and 0.543; 43.6, 67.3% and 0.489). The sensitivity, specificity, and AUC for MK after RFAwere 85.7, 96.7%, and 0.956 (Fig. 5a), respectively, which were significantly greater than those of MD (85.7, 83.3% and 0.839) (Fig. 5b) and ADC (78.6, 73.3% and 0.842; *P* < 0.05) (Fig. 5c) (Table 3).

**Table 3:** Predictive values of ADC, MK, and MD for pre-RFA and post-RFA

	AUC		95% CI		Youden index	Sensitivity	Specificity
	area	SE	lower limit	upper limit			
pre-ADC	0.560	0.099	0.366	0.735	0.176	0.443	0.533
post-ADC	0.842	0.062	0.720	0.913	0.519	0.786	0.733
pre-MD	0.543	0.094	0.360	0.726	0.210	0.512	0.567
post-MD	0.839	0.051	0.794	0.992	0.690	0.857	0.833
pre-MK	0.489	0.097	0.296	0.675	0.148	0.436	0.067
post-MK	0.956	0.030	0.697	0.987	0.824	0.857	0.967

Note-The sensitivity, specificity, and AUC for MK after RFA were significantly greater than those of MD and ADC

## Discussion

As a minimally invasive procedure, RFA is increasingly used to treat for early stage HCCs [14]. RFA carries low morbidity and mortality rates for HCC compared with hepatic resection, but the recurrence rate is higher [15]. So, it is important to identify a noninvasive method to predict the early recurrence of HCC treated by RFA. Study has pointed that HCC recurrence included metastatic spread and the emergence of de novo HCC [16], all which combined with the changes in the tumor microstructure. DWI can detect the properties of water molecule diffusion of Gaussian pattern. However, the diffusion motion of water molecules is referred to as non-Gaussian due to the existence of tissue barriers (e.g., cell membranes) and compartments (e.g. intracellular and extracellular spaces) in the human body. Compared with DWI, DKI sequences must set three or more b values and select a high b value, which can detect the properties of water molecule diffusion in tissues with non-Gaussian distribution and reflects the microscopic changes in tissues. DKI parameters include MK, MD, axial kurtosis, radical kurtosis, fractional anisotropy, and kurtosis anisotropy. These new metrics can better characterize the water diffusion properties, especially sensitive to diffusional heterogeneity [17].

The most commonly used DKI indexes in clinical studies are MK and MD. The extent of signal attenuation is shown by diffusion kurtosis. MK is considered as an evaluation criterion for the complexity of tissue microstructure. MK is closely related to tumor grade [18]. The complexity degree of the tissue structure within the area of interests is the main factor that influences the MK value. MD is the non-Gaussian distribution with corrected average ADC. The value only reflects the diffusion of water molecules. A study on breast cancer revealed that the values of MK and MD calculated by using the DKI model demonstrate remarkably higher specificity compared with ADC for the differentiation of benign and malignant breast lesions [19]. Yue et al. pointed that DKI has shown advantages in diagnosing and histologically grading endometrial cancer (EC) compared with conventional DWI [20]. In addition, the DKI correlation parameter (MK) was more sensitive to distinguish benign from malignant regions, as well as low- from high-grade malignant regions, within the peripheral zone (PZ) of the prostate than ADC [21]. MK may be a more meaningful indicator for the complexity of the organizational structure compared with MD [11]. The study on clear cell renal cell carcinoma showed that the MK displayed a better performance in distinguishing the normal renal parenchyma from clear cell renal cell carcinoma than MD [12]. All which studies were consistent with the study.

Many studies have used DKI to evaluate cerebral infarction, glioma, multiple sclerosis, Parkinson’s disease, and attention-deficit hyperactivity disorder [22–27]. Recently, mostdomestic and foreign scholars applied DKI for the exploratory of prostate, breast, and kidney cancers [28, 29, 12]. We evaluated the feasibility of this novel MR diffusion technique in the recurrence of early stage HCCs treated by RFA. We concluded that after RFA, the values of ADC and MD were higher; MK value was lower, which once again proved that RFA is an effective method for the treatment of early stage HCCs. In addition, the sensitivity, specificity, and AUC for MK after RFA were significantly greater than those of ADC and MD. Compared with DWI, DKI could quantify the actual diffusion of water molecules and reflect the microscopic changes in tissues, especially MK. Hence, DKI could objectively detect early stages of cancer to prevent the progression of HCC.

MK based on the DKI model may have better predictive accuracy in the prognosis of early stage single nodule HCCs after RFA and increased statistical power to reflect the microstructural complexity of the tumor compared with ADC derived from the conventional DWI model. The increased MK value may be due to the complex microenvironment of the tumor. The presence of multiple tissue types, such as tumor cell, necrosis, and inflammation, reflect more peaked distributions of tissue diffusivities in non-Gaussian diffusion behavior than in Gaussian diffusion behavior. Kurtosis of tissues has been reported to partially represent the interaction of water molecules with cell membranes and intracellular compounds. Study has described a significant decrease in MK in HCCs that were completely necrotic after RFA compared with those of background liver parenchyma [30]. The liver parenchyma is highly organized and contains many barriers for diffusion, such as liver cells, fibrous septa, and sinusoids. However, completely necrotic HCCs lose their cellularity, develop coagulation necrosis, and contain few diffusion barriers. An increased MK is remarkably correlated with the MVI of HCC. MVI-positive HCC is characterized by the presence of tumor emboli or mount of cancer cells in the branches of the vein and the spread and infiltration of tumor cells via microcirculation, which can limit the interaction of water molecules [31]. A related study revealed that the intercellular space decreases, cells are tightly aligned, and the volume of the cell nucleus increases with the exuberant cell proliferation of malignant tumors. Thus, DKI can detect early tumor growth and has high application value in predicting tumor recurrence.

The MK value, which is a significant parameter, allows preliminary parameter-based pre-operation pathological grading for patients with HCC and effectively predicts tumor recurrence. The values of MK are valuable for predicting recurrence in clinical trials for HCC.

Our study had several limitations. First, the relatively small population in our study might result in bias in the results. Second, RFA was performed by the percutaneous route, laparoscopic or open approach. Different ablation methods may affect the prognosis of HCCs. Third, the reference criteria for defining recurrent nodules is relatively weak.

## Conclusion

DKI is an independent predictor of the recurrence of early stage single HCCs treated by RFA. In addition, the prediction efficacy of MK is better than MD.

## Abbreviations

ADC: Apparent diffusion coefficient
AUC: Area under the curve
DCE: Dynamic contrast-enhanced
DKI: Diffusion kurtosis imaging
DWI: Diffusion weighted imaging
EC: Endometrial cancer
HCC: Hepatocellular carcinoma
MD: Mean diffusion
MK: Meankurtosis
MRI: Magnetic resonance imaging
MVI: Microvascular invasion
PZ: Peripheral zone
RFA: Radiofrequency ablation
ROI: Region of interest
TACE: Transcatheter arterial chemoembolization

## References

[1] Chen W, Zheng R, Baade PD, Zhang S, Zeng H, Bray F (2016). CA Cancer J Clin.

[2] Kim YS, Lim HK, Rhim H, Lee MW, Choi D, Lee WJ (2013). Ten-year outcomes of percutaneous radiofrequency ablation as first-line therapy of early hepatocellular carcinoma: analysis of prognostic factors. J Hepatol.

[3] Llovet JM, Ducreux M, Lencioni R, Di Bisceglie AM, Galle PR, Dufour JF (2012). EASL-EORTC clinical practice guidelines: management of hepatocellular carcinoma. J Hepatol.

[4] Shiina S, Tateishi R, Arano T, Uchino K, Enooku K, Nakagawa H (2012). Radiofrequency ablation for hepatocellular carcinoma: 10-year outcome and prognosticfactors. Am J Gastroenterol.

[5] Cui X, Wu Y, Wang Z, Liu X, Wang S, Qin C (2015). MicroRNA-34a expression is predictive of recurrenceafter radiofrequency ablation in early hepatocellular carcinoma. Tumour Biol.

[6] Lim KC, Chow PK, Allen JC, Chia GS, Lim M, Cheow PC (2011). Microvascular invasion is a better predictor of tumorrecurrence and overall survival following surgicalresection for hepatocellular carcinomacompared to the Milan criteria. Ann Surg.

[7] Goshima S, Kanematsu M, Kondo H, Yokoyama R, Tsuge Y, Shiratori Y (2008). Evaluating local hepatocellular carcinoma recurrence post-transcatheter arterial chemoembolization: is diffusion-weighted MRI reliable as an indicator?. J Magn Reson Imaging.

[8] Chen J, Zhang Y, Liang B, Yang Z (2010). The utility of diffusion-weighted MR imaging in cervical cancer. Eur JRadiol.

[9] Farley DR, Weaver AL, Nagomey DM (1995). “Naturalhistory”of unresected Cho-langio carcinoma: patient outcome after noncurative intervention. [J]. Mayo ClinProc.

[10] Yan C, Xu J, Xiong W, Wei Q, Feng R, Wu Y (2017). Use of intravoxel incoherent motion diffusion weighted MR imaging for assessment of treatment response to invasive fungal infection in the lung. [J]. EurRadiol.

[11] Jensen JH, Helpern JA, Ramani A, Lu H, Kaczynski K (2005). Diffusional kurtosis imaging: the quantification of non-gaussian water diffusion by means of magnetic resonance imaging. MagnReson Med..

[12] Dai Y, Yao Q, Wu G, Wu D, Wu L, Zhu L (2016). Characterization of clear cell renal cell carcinoma with diffusionkurtosis imaging: correlation betweendiffusion kurtosis parameters and tumorcellularity. NMR Biomed.

[13] Lencioni R, Llovet JM (2010). Modified RECIST (mRECIST) assessment for hepatocellular carcinoma. Semin Liver Dis.

[14] Shiina Shuichiro, Teratani Takuma, Obi Shuntaro, Sato Shinpei, Tateishi Ryosuke, Fujishima Tomonori, Ishikawa Takashi, Koike Yukihiro, Yoshida Haruhiko, Kawabe Takao, Omata Masao (2005). A Randomized Controlled Trial of Radiofrequency Ablation With Ethanol Injection for Small Hepatocellular Carcinoma. Gastroenterology.

[15] Cho YK, Rhim H, Noh S (2011). Radiofrequency ablation versus surgicalresection as primary treatment of hepatocellular carcinoma meeting the Milan criteria: a systematic review. J GastroenterolHepatol.

[16] Imamura H, Matsuyama Y, Tanaka E, OhkuboT HK, Miyagawa S (2003). Risk factors contributing to early and late phase intrahepatic recurrence of hepatocellular carcinoma after hepatectomy. J Hepatol.

[17] Jens HJ, Joseph A (2010). MRI quantification of non-Gaussian water diffusion by kurtosis analysis. Helpern. NMR Biomed.

[18] Zhu L, Pan Z, Ma Q, Yang W, Shi H, Fu C (2017). Diffusion kurtosis imaging study of rectal adenocarcinoma associatedwith histopathologic prognostic factors:preliminary findings. Radiology.

[19] Sun K, Chen X, Chai W, Fei X, Fu C, Yan X (2015). Breast cancer: diffusion kurtosis MR imaging—diagnostic accuracy and correlation with clinical-pathologic factors. Radiology.

[20] Yue W, Meng N, Wang J, Liu W, Wang X, Yan M (2019). Comparative analysis of the value of diffusion kurtosis imaging and diffusion-weighted imaging in evaluating the histological features of endometrial cancer [J]. Cancer Imaging.

[21] Rosenkrantz AB, Sigmund EE, Johnson G, Babb JS, Mussi TC, Melamed J (2012). Prostate cancer: feasibility and preliminary experience of a diffusional kurtosis model for detection and assessment of aggressiveness of peripheral zone cancer. Radiology.

[22] Assaf Y, Ben-Bashat D, Chapman J, Peled S, Biton IE, Kafri M (2002). High b value q-space analyzed diffusion-weighted MRI: application to multiple sclerosis. MagnReson Med.

[23] Raab P, Hattingen E, Franz K, Zanella FE, Lanfermann H (2010). Cerebral gliomas: diffusional kurtosis imaging analysis of microstructural differences. Radiology..

[24] Hori M, Fukunaga I, Masutani Y, Taoka T, Kamagata K, Suzuki Y (2012). Visualizing non-Gaussian diffusion: clinical application of q-space imaging and diffusional kurtosis imaging of the brain and spine. MagnReson Med Sci.

[25] Wang JJ, Lin WY, Lu CS, Weng YH, Ng SH, Wang CH (2011). Parkinson disease: diagnostic utility of diffusion kurtosis imaging. Radiology..

[26] Hori M, Fukunaga I, Masutani Y, Nakanishi A, Shimoji K, Kamagata K (2012). New diffusion metrics for spondylotic myelopathy at an early clinical stage. EurRadiol..

[27] Helpern JA, Adisetiyo V, Falangola MF, Hu C, Di Martino A, Williams K (2011). Preliminary evidence of altered gray and white matter microstructural development in the frontal lobe of adolescents with attention-deficit hyperactivity disorder: a diffusional kurtosis imaging study. J MRI.

[28] Barrett T, McLean M, Priest AN, Lawrence EM, Patterson AJ, Koo BC (2018). Diagnostic evaluation of magnetization transfer and diffusion kurtosis imaging for prostate cancer detection in a re-biopsy population. Eur Radiol.

[29] Christou A, Ghiatas A, Priovolos D, Veliou K, Bougias H (2017). Accuracy of diffusion kurtosis imaging in characterization of breast lesions. Br J Radiol.

[30] Goshima S, Kanematsu M, Noda Y, Kondo H, Watanabe H, Bae KT (2015). Diffusion kurtosis imaging to assess response to treatment in hypervascular hepatocellular carcinoma. AJR Am J Roentgenol.

[31] Wang WT, Yang L, Yang ZX, Hu XX, Ding Y, Yan X (2018). Assessment of microvascular invasion of hepatocellular carcinoma with diffusion kurtosis imaging. Radiology..

